# Variation of picture angles and its effect on the Concealed Information Test

**DOI:** 10.1186/s41235-020-00233-6

**Published:** 2020-07-31

**Authors:** Ann Hsu, Yu-Hui Lo, Shi-Chiang Ke, Lin Lin, Philip Tseng

**Affiliations:** 1 Graduate Institute of Mind, Brain and Consciousness, Taipei Medical University, No.250, Wuxin St., Taipei City, 11031 Taiwan; 2 Brain and Consciousness Research Center, Shuang Ho Hospital, Taipei Medical University, New Taipei City, Taiwan; 3 Psychiatric Research Center, Wan Fang Hospital, Taipei Medical University, Taipei, Taiwan

**Keywords:** Guilty knowledge test, Concealed Information Test, Concealed knowledge test, Deception, Memory detection, Recognition memory

## Abstract

**Background:**

The reaction time-based Concealed Information Test (RT-CIT) is a memory paradigm used to detect crime-related knowledge. However, this would also imply that the RT-CIT would be vulnerable to factors that are known to compromise object recognition or memory integrity. From this perspective, one key issue is whether “guilty” memory can be detected if the crime-related images are photographed at different angles from what the perpetrator saw, which is almost always the case in the field. To investigate this, here we manipulated the deviation angles, from 0° to 330° in 11 steps, between the study and test phases to assess how the RT-CIT holds up against angular rotations.

**Results:**

We observed a robust RT-CIT effect at all deviation angles for both deep-encoders (Experiment 1) and shallow-encoders (Experiment 2). The RT-CIT was effective within the first 250 or so trials for both encoding groups, with smaller probe-irrelevant differences beyond that.

**Conclusions:**

With appropriate encoding and memory strength, RT-CIT images do not necessarily have to match the exact angle of the perpetrator’s perspective at the time of the crime. Unnatural angles such as 90° and 270° or unconventional rotational axes may require caution. Trial number under 250 trials show maximal Probe-Irrelevant difference, but more trials may add power to improve detection accuracy.

## Significance

The reaction time-based Concealed Information Test (RT-CIT) is a memory task that detects one’s reaction time cost when viewing a familiar photograph. Thus, the guilty should react slower to crime-related pictures relative to pictures not from the crime. The RT-CIT, however, relies heavily on suspects’ memory integrity and is therefore potentially susceptible to factors that are known to impair memory integrity or interfere with memory-matching capability. Here, we tested one possible weakness of the RT-CIT: namely, how accurate (and resemblant to suspects’ memory) do the relevant pictures have to be? Our participants saw only one viewpoint of a probe object, but were tested with images of 12 different angles of the same probe item. To our surprise, we found the RT-CIT to be quite resistant to picture rotations across all angles, and this was true for both deep-encoding (Experiment 1) and shallow-encoding (Experiment 2) conditions. Furthermore, for deep-encoders, the RT-CIT was most effective under 250 trials, but remained accurate within almost 800 trials. We conclude that, for well-remembered items or pictures, the forensic team does not need to worry about reconstructing the angles (e.g., approximate the eye height and tracing the footsteps of the perpetrator) that closely match what the perpetrators saw.

## Background

The Concealed Information Test (CIT) is a method that aims to identify suspects who purposely deny knowledge of a specific crime (for a review, see Ben-Shakhar, 2012). The CIT has several widely-used versions in research: one based on polygraph recordings, and the others based on reaction time (RT) or electroencephalogram (EEG) (although combined usage with other neuroimaging or physiological measurements is also possible). The polygraph-based CIT relies on analyzing the suspects’ physiological responses while they hear questions regarding the crime that only the guilty would know the answer to. For example, the suspect may hear questions like “was the stolen jewel, a diamond ring?” or “if you were the thief, you would know that the stolen jewel was one of the following items: A) a watch, B) a ring, C) a necklace, and D) a pin”. The rationale is that the innocent, no matter how anxious they may appear on the polygraph, could not recognize key items that are specific to a crime if they have not participated in or read anything about the crime. Thus, only the culprit would be physiologically more responsive to words or pictures that he/she can remember from the crime, thereby producing a larger physiological response on the polygraph (Lykken, 1959).

The RT and EEG-based CIT also emphasizes recognition memory. It targets automatic memory recognition (and the active suppression that follows) in suspects who deny guilty knowledge, coupled with EEG or RT (for a meta-analysis and review, see Meijer, Selle, Elber, & Ben-Shakhar, 2014). In this version of the CIT, participants are to provide truthful yes/no responses to a series of photographs for Target information that they recognize (e.g., picture of their friend) or Irrelevant information that they do not recognize (e.g., picture of a stranger), respectively. Crucially, crime-related stimuli (i.e., Probes) are inserted intermittently among the Targets and Irrelevants, which the suspects recognize but are motivated to suppress recognition of and respond “no” as if the stimuli are Irrelevants. Using the Target as the baseline for recognized information and the Irrelevant as the baseline for unrecognized information, (Farwell and Donchin 1991) demonstrated that the guilty would show higher amplitude of the P300 component in the EEG upon seeing the Probes, which implies recognition. Furthermore, Seymour and colleagues also showed that a similar detection rate can be achieved by analyzing the participants’ reaction time data alone even without use of the EEG (Seymour & Kerlin, 2008; Seymour, Seifert, Shafto, & Mosmann, 2000). This is because the automatic activation of recognition memory, as well as the active suppression that follows it, introduce extra cognitive stages of processing that take time, which are enough to produce notable delays in the RT-based CIT (for a review of the RT-based CIT, see Verschuere, Suchotzki, & Debey, 2015). Indeed, one recent meta-analysis of 114 studies (34 of which were RT-based CIT studies) reported a large standardized RT difference between crime-relevant Probes and crime-irrelevant stimuli, even after correction for publication bias (Suchotzki et al., 2017).

The RT-based CIT (referred to as RT-CIT hereafter) is essentially a recognition memory paradigm designed to detect crime-related memory traces. This is drastically different from the polygraph and the Control Question Test (CQT), which uses a combination of skin conductance, breathing, and heart rate to measure and contrast arousal levels associated with truth-telling (Irrelevant question such as “Is your name John Smith”) and deception (Control question such as “Have you ever verbally threatened to hurt anyone” vs Relevant question such as “Did you assault Jane Smith the evening of April 11th”). As such, the CIT is preferred over the polygraph (Elaad, 1990). The RT-CIT, however, is not without its limitations and weaknesses. For example, its reliance on memory over guilt suggests that participants who have witnessed the crime on video, instead of actually committing it, can also fail to pass the RT-CIT (Bradley, Barefoot, & Arsenault, 2011; Lukács & Ansorge, 2019). Therefore, the RT-CIT does not differentiate the guilty from the innocent, only the informed from the non-informed, which places conditional constraints on its uses in the field (for a possible remedy of this issue, see Lukács & Ansorge, 2019).

## The current study

Due to the memory-matching nature of the RT-CIT, it makes theoretical sense that any factors that may reduce the *resemblance* between test stimuli and one’s memory representations of the Probes can also potentially compromise the RT-CIT. One such example is the angle from which the Probe is photographed, and is the focus of the current study. That is, can the RT-CIT still be effective when the picture of the crime-related items is photographed from a different angle or perspective from that which the perpetrator saw (or remembers)? This problem is not uncommon in the field since the forensic team does not always have information (e.g., security footage) regarding the culprit’s gaze and head angle at the scene. Thus, relevant RT-CIT images can often be photographs of Probe items laid flat against the ground from a 180° angle in a two-dimensional manner, which may not be the way the culprit perceived or remembered the same item. Therefore, the answer to this question would have far-reaching implications for how the forensic team should photograph the Probe items at the scene of the crime.

At the heart of this issue is the longstanding debate of whether object recognition (and, hence, memory detection) is viewpoint-dependent or viewpoint-invariant (e.g., Tarr, Williams, Hayward, & Gauthier, 1998). Based on the classic findings from Shepard and Metzler’s (1971) mental rotation study where participants’ RT for same/different judgment increased linearly with the amount of angular rotation between two objects, there is empirical support to suspect that this may be the case in the RT-CIT. In addition, by manipulating the deviations between study and test angles of geons, Tarr et al. (1998) also found that object memory matching became increasingly more difficult as the difference between studied and tested viewpoints increased. Crucially, this effect of viewpoint-dependence is largest when recognition has to be object-specific instead of categorical (Hamm & McMullen, 1998), which raises concern for RT-CIT validity because we do not want the culprit to just recognize a superordinate category (e.g., gun), but a specific sample within that category (e.g., the gun I used last Friday).

Taken together, if object representation in memory is viewpoint-dependent, then the Probe–Irrelevant RT difference may decrease as a function of increasing dissimilarity between the Probe photograph and what the culprit actually saw. In this case, there would likely be an upper limit of the angular difference that, if exceeded, would make a guilty suspect appear innocent (non-significant RT difference between Probe and Irrelevant). Findings from the object recognition literature seem to suggest this critical angle to be within the range of 60°–120° (Jolicoeur, 1985, 1990; Jolicoeur, Regehr, Smith, & Smith, 1985). If so, this would imply that the forensic team should follow the footsteps of the culprit closely and approximate their height when taking pictures of the Probe items in order to provide a close match. However, if angular differences do not significantly affect the RT gap between Probe and Irrelevant responses, then perhaps the RT-CIT is robust against angularly-mismatched photographs, and thus no additional guidelines are needed when photographing the Probe items. To answer this question, in this study we manipulated the deviation angles, from 0° to 330° in 11 steps, between the pictures that were seen initially and the ones that were eventually used for the RT-CIT.

### Methods

#### Participants

Twenty-seven participants (12 male, 15 female; age 20–35 years, mean age = 24.03) with normal eyesight or corrected-to-normal eyesight were recruited for this experiment. All participants gave informed consent prior to their participation, and received financial compensation for their time. Three participants’ data were excluded from analysis due to low accuracy in the main task. All experimental procedures were approved by the Joint Institutional Review Board of Taipei Medical University, Taiwan.

#### Apparatus and materials

Study and test materials were images of carefully selected shoes (matched based on similar color, function, shape, etc.) across Targets, Probes, and Irrelevants. The entire experiment used a total of 60 shoes. During the design phase, we randomly chose 10 shoes to serve as Probes, then specifically selected another 10 that matched the Probes in appearance/function to serve as Targets, and used the remaining 40 as Irrelevants. These 10 Probes, 10 Targets, and 40 Irrelevants were then used as experimental stimuli for all participants (see Supplementary Figure).

A total of 720 unique images were made out of these 60 shoes: namely, for every shoe, 12 images were rendered from the 360*°* rotation view, starting from 0° (i.e., tip of the shoe facing directly to the right), in increments of 30*°* (i.e., 0°, 30°, 60*°*, 90°, 120*°*, 150*°*, 180*°*, 210*°*, 240*°*, 270*°*, 300*°*, 330*°*)*.* This resulted in a total of 120 Probe images, 120 Target images, and 480 Irrelevant images.^1^

One angle per item (i.e., 10 Probes and 10 Targets) was selected to be the angle that the participants looked at during the studying phase (i.e., 20 images in total; see Supplementary Figure). These randomly selected images of Targets and Probes were then used for all participants, and became the 0*°* basis for computing deviation angles for further analysis.

The ratio of the Target, Probe, and Irrelevant stimuli in the experiment was 1:1:4 (e.g., Verschuere & De Houwer, 2011). The present study used a multiple-probe protocol using 10 Probe, 10 Target, and 40 Irrelevant shoes (for a comparison between single-probe and multiple-probe protocols, see Verschuere, Kleinberg, & Theocharidou, 2015).

#### Design and procedure

The experiment consisted of two phases: a study phase (for Target and Probe items) and a test phase.

##### Study phase

Participants first viewed and memorized images of 10 Target items and 10 Probe items, and were explicitly told that they would later be tested and must suppress their memory/knowledge of the Probe items. For each category (Target vs Probe), all 10 items were of a different angle, thus covering 10 out of 12 angles, and 90*°* and 270*°* were excluded from the study phase due to their high level of difficulty (but were included in the test phase)*.*

To ensure vivid memory of all Target and Probe items, participants went through three different tasks during the study phase. During the study phase, participants first filled out a questionnaire regarding each of the Target and Probe items. They were asked to identify the category, colors, and materials of the item in each image, as well as answering questions regarding how much the shoe in the picture appealed to them, how fashionable they thought it was, how much they thought the retail price would be, and how much they would be willing to pay for it. This was done for all 20 images (approximately 15 min), which was designed to motivate the participants to pay close attention to various details of the Target and Probe items. After the questionnaire, participants were given jigsaw puzzles to complete, one at a time, of all 10 Target images, followed by all 10 Probe images (in the exact same angle as the study session). This task was designed to encourage the participants to encode the holistic structure of the items, and the average time spent in this section was also about 15 min. Again, these activities were done in order to increase the different aspects of visual impression of these stimuli and increase their memory strength for subsequent testing.

Lastly, before going into the main test, participants completed one last pretest. In this pretest, one image at a time was presented (10 Target, 10 Probe, and 20 new images) on display for 5 s in randomized order, and participants were to press “F” for Target and “J” for Probe, or the spacebar for new images that they have not seen before. The response time had to be less than 5 s to be scored as accurate. At the end of each trial there was immediate feedback on display as well as their cumulative overall accuracy (500-ms duration). This pretest was conducted twice for all participants.

Between the first and second pretests, participants performed one final rehearsal with a deck of 20 flashcards of the studied Target and Probe items. Participants were to shuffle the deck and speak out that they “have seen it before” or “have not seen it before” upon seeing Target and Probe items, respectively. The average duration for the final rehearsal was around 5 min.

**Fig. 1:**
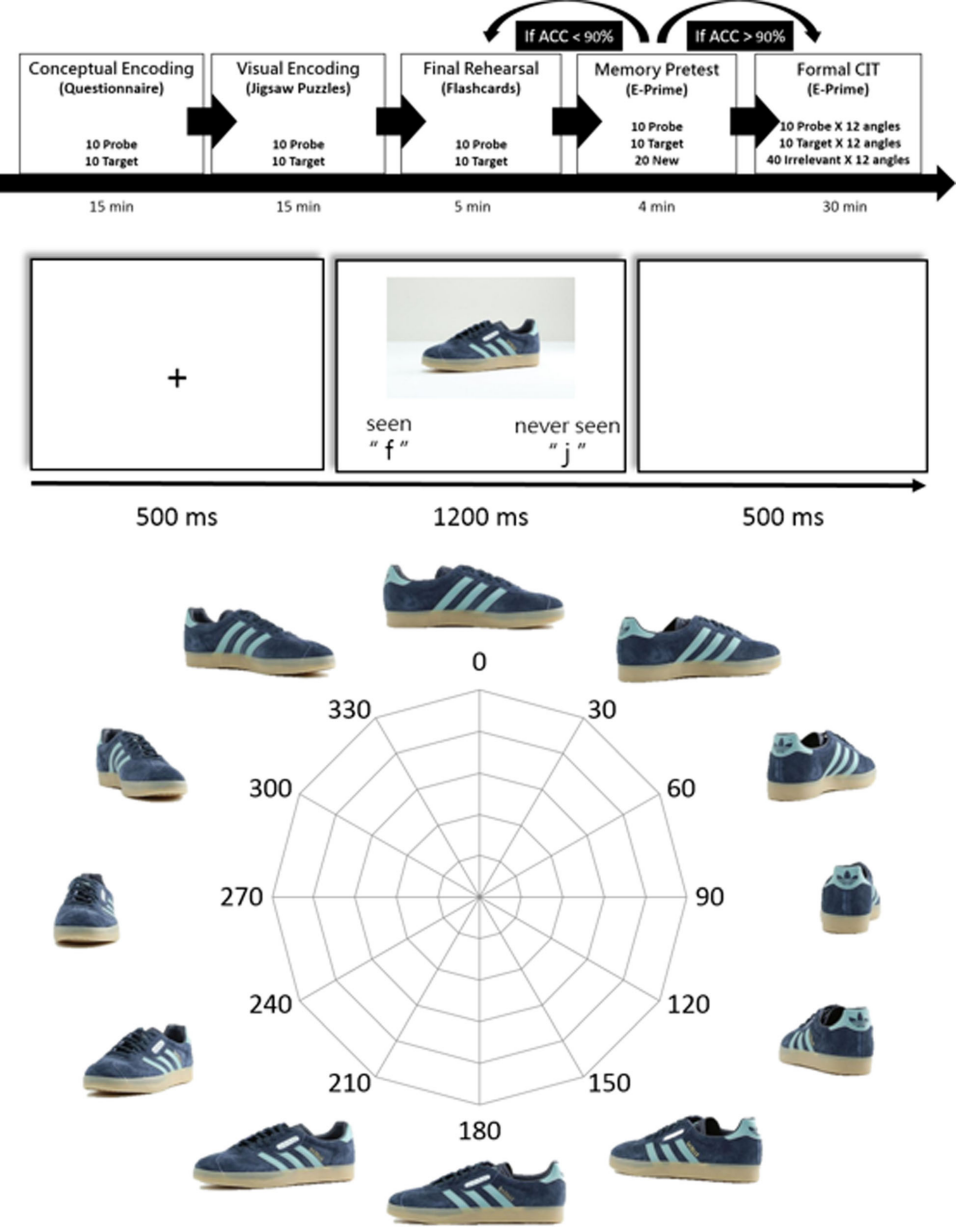
Study design. Top panel: procedure of the study phase and test phase. To ensure proper encoding and memory strength, participants went through three training tasks (i.e., questionnaire, jigsaw puzzle, and flashcard), and were then tested for their memory in the pretest. Participants who completed the pretest with accuracy above 90% would continue to perform the formal reaction time-based Concealed Information Test (RT-CIT). Those with accuracy below 90% had to go back to the flashcard task, and then perform the pretest again until their pretest accuracy was over 90%. Middle panel: trial procedure in the test phase. Note that the actual display of “seen” and “never seen” (or “have not seen”) in the experiment was written in Traditional Chinese. Bottom panel: example of 12 angular rotations of one item. The presentation angles are defined such that the inward profile view is always 0°, and so on. During the study phase, participants saw only one view, and were tested with all views later in the test phase

At the end of the pretest, only participants who have correctly categorized 36 out of all 40 images (90% accuracy) from the pretest were admitted into the formal test phase. This was done to ensure that both Targets and Probes were well encoded into participants’ memory with minimal confusion. Participants who scored under 90% accuracy had to restudy the flashcards and redo the pretest until they achieved at least 90% accuracy (Fig. 1, top).

Note that although participants completed extensive training during the study phase, they have only seen one angle for each Target or Probe item. That is, the same Target and Probe images were used repeatedly throughout the memorization period, jigsaw puzzle, and pretest. Therefore, although the participants were very familiar with every Target and Probe item (from a particular angle) thus far studied, there were 11 other versions, or angles, of the same Target and Probe item that they had not seen before, until the test phase.

##### Test phase

During the formal experimental test phase, there were 760 trials in total, which came from 140 Target trials (all 12 angles for 10 items, plus one repeating trial for 0*°* and 180*°* per item), 140 Probe trials (all 12 angles for 10 items, plus one repeating trial for 0*°* and 180*°* per item^2^), and 480 Irrelevant trials (all 12 angles for 40 items). All trial types and all angle deviations were intermixed in randomized order. At the start of every trial, a 500-ms fixation cross was displayed at the center, followed by an image with a duration of 1200 ms, and ended with a 500-ms inter-trial interval (Fig. 1, middle). Participants were to press “F” for Target, “J” for Irrelevant, and again “J” for Probes that they recognized but were instructed to conceal. Participants were instructed to react as fast as possible while being highly accurate. A break was provided every 60 trials.

In the test phase, participant(s) with low accuracy in the Target condition would be excluded from further data analysis. Although low accuracy in Targets is typically observed in the RT-CIT due to Targets’ low probability of occurrence, an unusually low Target accuracy (< 50%; Kleinberg & Verschuere, 2015) may indicate either poor learning of the Targets or a deliberate attempt to say “no” for every item. To this end, we set the exclusion criterion for Target accuracy at 1.5 grand SD (i.e., 52.80% accuracy) below the grand mean Target accuracy, which resulted in the exclusion of three participants (Target accuracy: 42.37%, 45.00%, and 47.14%).

### Results

We first analyzed the accuracy and RT between Probe, Irrelevant, and Target trials without considering the angles by collapsing all trials of different angular rotations together.^3^ This gives us the traditional RT-CIT comparisons. Furthermore, error trials with wrong or no responses were discarded from analysis of RT data.

**Fig. 2:**
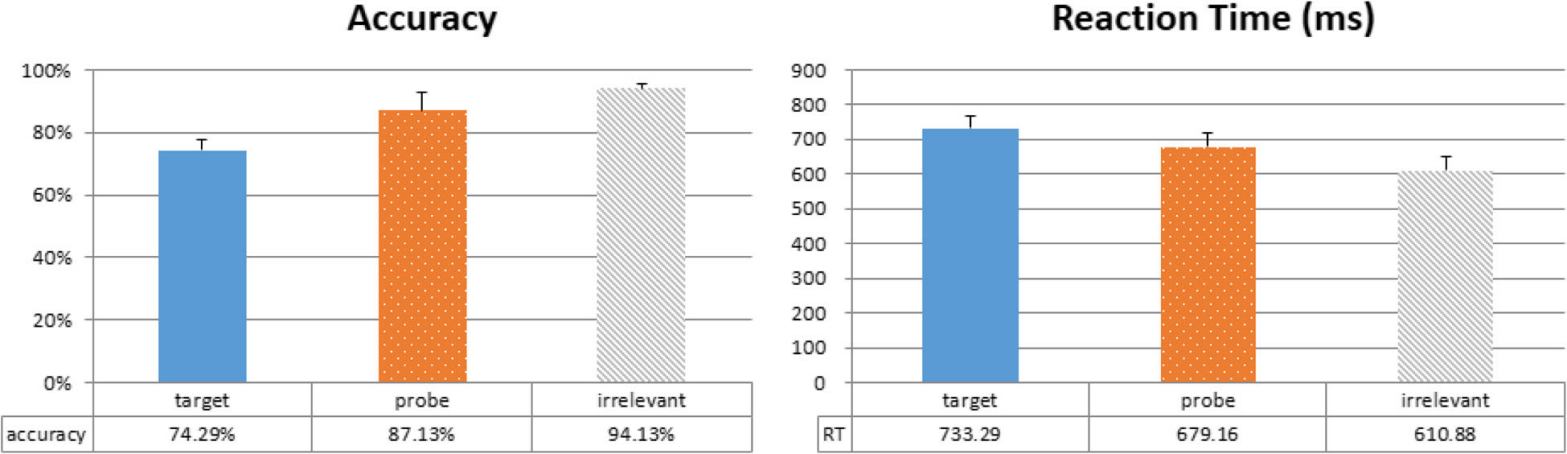
Overall accuracy and reaction time. Probe item accuracy was lower than those of Irrelevant items, and the reaction time was higher. These numbers suggest a reliable reaction time-based Concealed Information Test effect when all angles are collapsed together

In terms of RT, a repeated-measures one-way ANOVA (Probe, Target, Irrelevant) showed a significant main effect of trial type, *F*(2,46) = 81.236, *p* < 0.001, η^2^_p_ = 0.779. Subsequent post-hoc comparisons using Bonferroni correction revealed significant differences between RT from all trial types: Target vs Probe, *t*(23) = 5.934, *p* < 0.001, *d* = 1.211; Target vs Irrelevant, *t*(23) = 11.758, *p* < 0.001, *d* = 2.400; and Probe vs Irrelevant, *t*(23) = 7.350, *p* < 0.001, *d* = 1.500 (Fig. 2, right). Critically, the significant difference between Probe and Irrelevant replicates the classic RT-CIT finding, and our observed RT difference is in the same range as previous reports (Noordraven & Verschuere, 2013; Verschuere, Kleinberg, et al., 2015). Similar observations were also made in terms of accuracy (Fig. 2, left). A repeated-measures one-way ANOVA revealed a significant main effect of trial type, *F*(2,46) = 28.185, *p* < 0.001, η^2^_p_ = 0.551, where post-hoc comparisons with Bonferroni correction also showed significant differences between all trial types: Target vs Probe, *t*(23) = − 3.745, *p* = 0.002, *d* = − 0.764; Target vs Irrelevant, *t*(23) = − 9.862, *p* < 0.001, *d* = − 2.013; and Probe vs Irrelevant, *t*(23) = − 2.919, *p* = 0.008, *d* = − 0.596. Together, these results highlight the robustness of the RT-CIT, and suggest that our participants do take longer to deny recognition of a previously-seen object. In particular, Target trials have lower accuracy and longer RT, which is also similar to previous reports. This is perhaps due to the unequal 1:5 probability of yes and no responses (Target vs Probe and Irrelevant) that has biased most participants to use “no” as their default response.

#### Effect of study-test angular deviations

To investigate the possible effect of angle differences between the study and RT-CIT sessions, angular deviation from 0° to 360° was analyzed symmetrically such that any given deviation can come from two rotations. For example, if a given item was seen at 120° (i.e., presentation angle), an angular deviation of 30° can be obtained bidirectionally from the same item photographed at 150° and 90°, or a deviation of 60° from the 180° and 60° images, and so on. Thus, although there are 12 different presentation angles (0°–360°, whose effect is presented in the following section), there are only six angular deviations (30°–180°). It is worth emphasizing again that the present study reports the effects of two kinds of angles, *presentation* and *deviation*, although we are primarily interested in the latter (e.g., seeing a shoe at a 30° presentation angle and later being tested using a 90° presentation angle would yield a study-test deviation angle of 60° in our analysis).

**Fig. 3:**
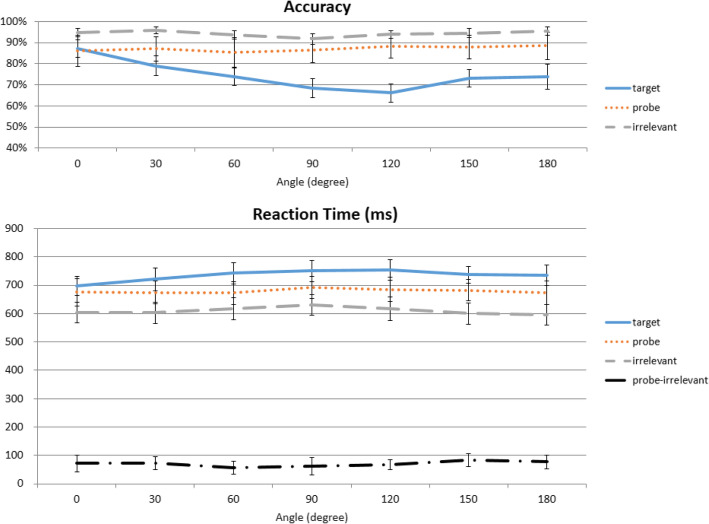
Effect of angular deviations. Participants were tested on the same (0°) or rotated versions of what they saw (30°–330°, symmetrically collapsed into 30°–180° here). The flat orange line shows no effect of angular deviation on Probe reaction times (RTs), but they nonetheless are slower than Irrelevant RTs at all angles

In accuracy, a repeated-measures two-way ANOVA with the factors of trial type (Probe, Target, Irrelevant) and angular deviation (0°, 30°, 60°, 90°, 120°, 150°, 180°) revealed a main effect of both trial type, *F*(2,46) = 27.830, *p* < 0.001, η^2^_p_ = 0.548, and deviation, *F*(6,138) = 12.434, *p* < 0.001, η^2^_p_ = 0.351, as well as a significant interaction between the two, *F*(12,276) = 9.564, *p* < 0.001, η^2^_p_ = 0.294. Subsequent one-way ANOVAs showed that there were significant effects of angular deviations for Targets, *F*(6,138) = 15.833, *p* < 0.001, η^2^_p_ = 0.408, and Irrelevants, *F*(6,138) = 6.250, *p* < 0.001, η^2^_p_ = 0.214, but not for Probes, *F*(6,138) = 0.857, *p* = 0.561, η^2^_p_ = 0.036. These results indicated that trial type was an important factor, but no effect of angular deviation was detected for Probe items (Fig. 3).

Importantly, is the RT-CIT’s effectiveness compromised by differing angles between the study and test phases? A 3 × 7 repeated-measures ANOVA on participants’ RT data showed a significant effect of trial type, *F*(2,46) = 84.899, *p* < 0.001, η^2^_p_ = 0.787, and deviation, *F*(6,138) = 7.455, *p* < 0.001, η^2^_p_ = 0.245, but no interaction between the two, *F*(12,276) = 1.871, *p* = 0.080, η^2^_p_ = 0.075. To help interpretation of the null interaction, a Bayesian repeated-measures ANOVA was performed using the open-source software package JASP (Wagenmakers et al., 2018). The main effect of trial type was significant, reflected in a higher Bayes factor for the alternative hypothesis (H_1_: trial type influences participants’ RT; BF_10_ = 6.467 × 10^84^) than for the null hypothesis. In contrast, the Bayes factor for the effect of angular deviation was less than one (BF_10_ = 0.121). For the interaction, we compared the BF_10_ value for the model with interaction against the BF_10_ value for the model with only two main effects: the Bayes factor for the interaction term was 0.058, providing strong evidence for no interaction (17.367 times more likely than the alternative hypothesis).

Looking at the Probe RTs across all angles it is quite apparent that the RT distribution is flat across all angles, which is reflected by the absence of a significant one-way ANOVA, *F*(6,138) = 0.724, *p* = 0.575, η^2^_p_ = 0.031. Also, none of the paired-sample *t* tests between every Probe angle reached statistical significance. Most importantly, is the Probe–Irrelevant difference altered by angular rotations? To answer this question, we computed the RT differences between Probe and Irrelevant conditions across every angle, and submitted them to a one-way repeated-measure ANOVA. The RT differences between Probe and Irrelevant were flat across all angles, which was reflected by the absence of a significant one-way ANOVA, *F*(6,138) = 0.942, *p* = 0.467, η^2^_p_ = 0.039 (Fig. 3). Separate comparisons also showed that Probe RTs are significantly slower than their Irrelevant counterparts at every angle: 0°, *t*(23) = 4.884, *p* < 0.001, *d* = 0.997; 30°, *t*(23) = 6.343, *p* < 0.001, *d* = 1.295; 60°, *t*(23) = 4.728, *p* < 0.001, *d* = 0.965; 90°, *t*(23) = 3.980, *p* = 0.004, *d* = 0.812; 120°, *t*(23) = 7.645, *p* < 0.001, *d* = 1.561; 150°, *t*(23) = 6.955, *p* < 0.001, *d* = 1.420; and 180°, *t*(23) = 6.309, *p* < 0.001, *d* = 1.288 (Bonferroni correction). Therefore, these data suggest that the Probe RT remains significantly slower than the Irrelevant RT in a fairly consistent manner, and that we could not detect an effect of angular deviations.

#### Effect of presentation angle

**Fig. 4:**
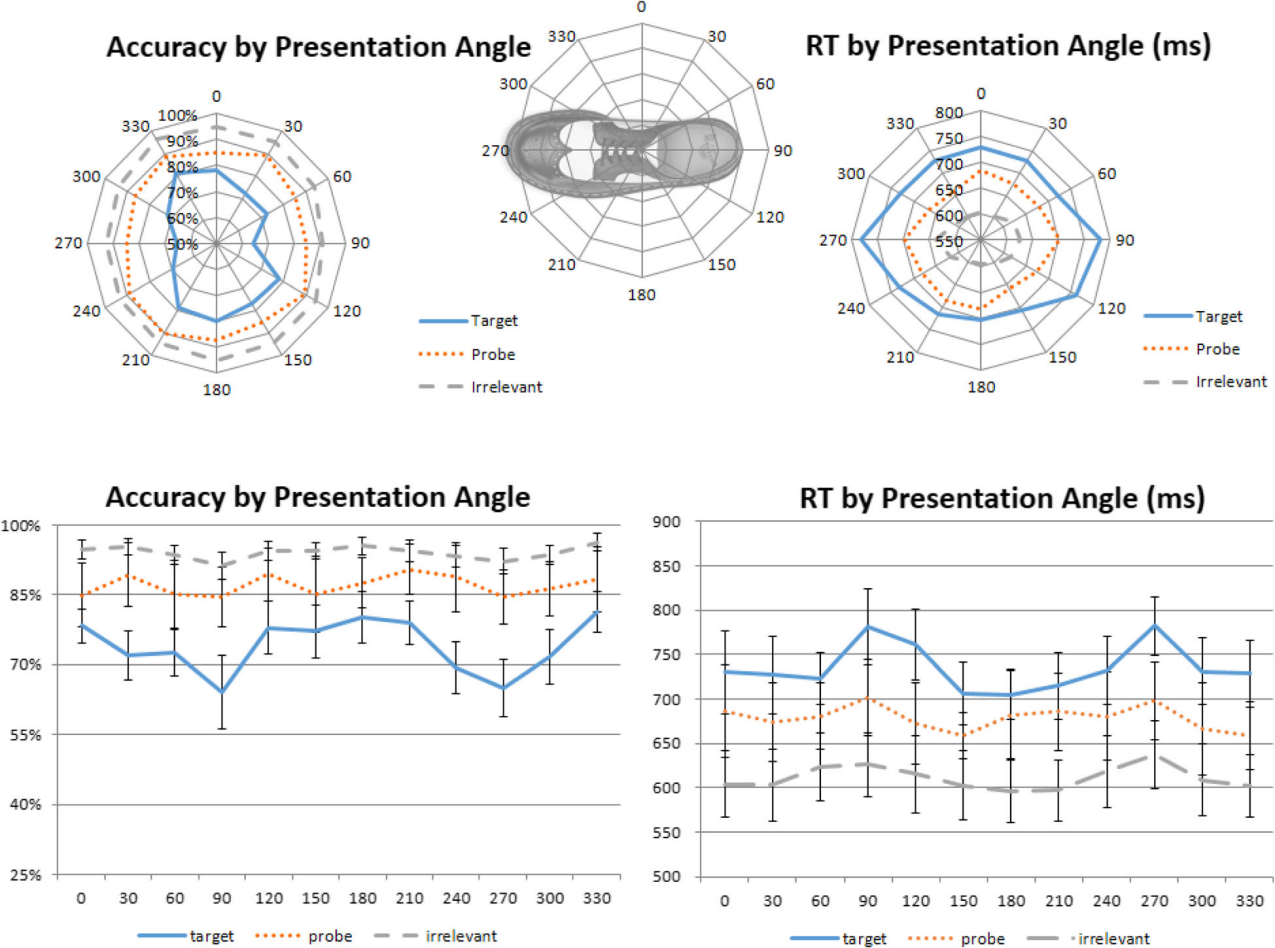
Effect of presentation angle. Top panel: radar graphs depicting the effects of the presentation angles on accuracy (left) and reaction time (RT) (right). Lower panel: a strong presentation angle effect in Targets and a mild effect in Irrelevants, but no effect in Probes

Apart from deviations in angle rotations, some viewing angles of an object may naturally be more difficult than others because they provide less visual cues or information regarding object identity. For example, in the context of this experiment, images from the 90° and 270° categories might be much harder to identify due to the least amount of information that they contain (Fig. 4, top), which was why these angles were excluded from the study phase (but were used in the test phase to ensure that all deviation angles were covered). Although these angles would be stimulus-specific, and are not the main focus of the present study, it is important to consider whether some angles may require caution for a particular set of Probe images. To this end, to explore whether there are any differences in levels of difficulty that are naturally associated with a specific image angle, here we tested whether some angles might be harder than others for the participants to recognize, regardless of angular deviation.

In both accuracy and RT, a two-way repeated-measures ANOVA with the factors of trial type (Target, Probe, Irrelevant) and presentation angle (12 angles from 0° to 330°) showed a main effect for both trial type (accuracy, *F*(2,46) = 28.691, *p* < 0.001, η^2^_p_ = 0.555; RT, *F*(2,46) = 83.368, *p* < 0.001, η^2^_p_ = 0.784) and presentation angle (accuracy, *F*(11,253) = 8.807, *p* < 0.001, η^2^_p_ = 0.277; RT, *F*(11,253) = 5.910, *p* < 0.001, η^2^_p_ = 0.204), as well as a significant interaction between them in accuracy, *F*(22,506) = 3.224, *p* = 0.004, η^2^_p_ = 0.111, but not a statistically significant interaction in RT, *F*(22,506) = 1.207, *p* = 0.310, η^2^_p_ = 0.050.

We then conducted a trend analysis for each trial type. For accuracy, there were significant quartic trends for Target and Irrelevant items, but not Probe items (Target, *F*(1,23) = 38.903, *p* < 0.001, η^2^_p_ = 0.628; Irrelevant, *F*(1,23) = 9.456, *p* = 0.005, η^2^_p_ = 0.291; Probe, *F*(1,23) = 0.919, *p* = 0.348, η^2^_p_ = 0.038). For RT, significant quartic trends were observed for both Target and Irrelevant items, but not Probes (Target, *F*(1,23) = 16.191, *p* = 0.001, η^2^_p_ = 0.413; Irrelevant, *F*(1,23) = 37.670, *p* < 0.001, η^2^_p_ = 0.621; Probe, *F*(1,23) = 2.182, *p* = 0.153, η^2^_p_ = 0.087). These results confirm our initial suspicion that perhaps the 90° and 270° angles were more difficult to detect than others. Thus, police investigators may wish to consider avoiding those angles in forensic contexts. Furthermore, there seems to be a weak quartic trend in Probe RT as well (Fig. 4, right) but, perhaps due to the number of trials, such a trend was not statistically significant.

#### RT-CIT efficacy over time

**Fig. 5:**
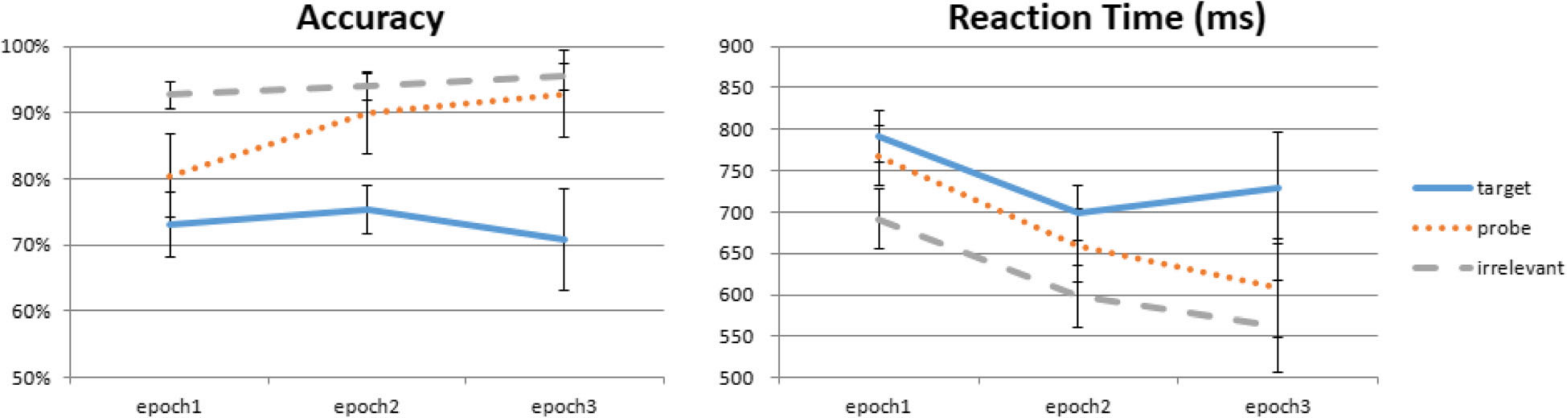
RT-CIT efficacy over time. Although participants are seeing Probe items from different angles, multiple exposures over time may still help the participants build a richer mental representation and potentially compromise the reaction time-based Concealed Information Test (RT-CIT). Here, the gap between Probe and Irrelevant is biggest in the first 253 trials, gradually decreasing as the number of exposures cumulates

Lastly, one follow-up question was whether the RT-CIT’s effectiveness would decrease as participants gained more exposure to the different rotated versions (11 total) of the Probe image. It seems that multiple exposures, although of photographs from different angles, would nonetheless still facilitate a more complete mental representation of the Probe item. To investigate this, we divided the 760 trials into 3 epochs by time (i.e., first 253, middle 253, and last 254 trials). A repeated-measures ANOVA on RT data with factors of trial type (Probe, Target, Irrelevant) and epoch (1, 2, 3) revealed main effects of both epoch, *F*(2,46) = 19.353, *p* < 0.001, η^2^_p_ = 0.457, and trial type, *F*(2,46) = 67.252, *p* < 0.001, η^2^_p_ = 0.745, as well as a significant interaction between them, *F*(4,92) = 5.265, *p* = 0.014, η^2^_p_ = 0.186. To further explore the relationship between Probe and Irrelevant stimuli, post-hoc analysis with Bonferroni correction suggest that the interaction was driven by the closing gap between the Probe and Irrelevant RTs from the first, second, and third epochs, which was due to a steeper decrease of RT for the Probes (first, *t*(23) = 6.504, *p* < 0.001, *d* = 1.328; second, *t*(23) = 6.364, *p* < 0.001, *d* = 1.299; third, *t*(23) = 3.617, *p* = 0.004, *d* = 0.738; see Fig. 5, right). However, even at the third epoch the Probe–Irrelevant RT difference was statistically significant, once again validating the robustness of the RT-CIT. These results suggest that, although participants were becoming faster in Probe RT, in the context of ~ 800 trials the RT-CIT is good enough to still separate Probe from Irrelevant trials.

In contrast, the accuracy measure from the RT-CIT is more susceptible to multiple exposures of Probe images over time. In a two-way ANOVA, we observed a main effect of trial type, *F*(2,46) = 30.183, *p* < 0.001, η^2^_p_ = 0.568, and epoch (1, 2, 3), *F*(2,46) = 4.749, *p* = 0.021, η^2^_p_ = 0.171, and a significant interaction between them, *F*(4,92) = 4.738, *p* = 0.012, η^2^_p_ = 0.171. The interaction was also driven by the rapidly improving accuracy of Probe items (Fig. 5, left). Post-hoc analysis with Bonferroni correction showed that Probe accuracy went from ~ 80% in the first epoch to ~ 90% in the second epoch and ~ 93% in the third epoch (first, *t*(23) = − 4.208, *p* = 0.001, *d* = − 0.859; second, *t*(23) = − 1.691, *p* = 0.313, *d* = − 0.345; third, *t*(23) = − 0.943, *p* = 1.000, *d* = − 0.193). Therefore, unlike the robust effect in RT, there was no difference between Probe and Irrelevant accuracy after the first ~ 250 trials.

### Discussion

In this experiment we investigated whether angle differences between RT-CIT photographs and participants’ actual exposure and memory would have an impact on RT-CIT accuracy. To our surprise, we could not detect an effect of angular rotations. Therefore, although the participants have never seen 11 out of the 12 angles used here, they nonetheless were able to recognize the Probe items and show significant RT difference between Probes and Irrelevants at all angular rotations (Fig. 3, lower panel). In addition, we observed a significant presentation angle effect at 90° and 270°, and that the slower RT of Probes decreased over time but remained significantly different from Irrelevants at almost 800 trials.

The absence of a detectable angular effect in the RT-CIT is good, but also puzzling. Most studies to date have supported the viewpoint-dependent view (e.g., Riesenhuber & Poggio, 2000). Particularly, the classic Shepard and Metzler (1971) study was the first to show a linear relationship between angular rotation and recognition RT. However, since our participants here were comparing one displayed photograph with others stored in their memory, it is plausible that such a process may be different from the online, juxtaposed picture-comparison paradigm that is often used in object recognition studies. In other words, the culprit here may be the different processes involved in memory-based (e.g., RT-CIT) and perception-based (e.g., object recognition literature) comparisons.

One possible factor that is critical in memory-based comparisons, but absent in perception-based comparisons, is memory strength, or depth of encoding. In the context of the RT-CIT, one important study by Seymour and Fraynt (2009) has previously investigated the role of encoding strength (and its possible interaction with memory decay time). These authors randomly assigned participants into either a deep or shallow probe-study condition, as well as three different delay conditions: 10 min, 24 h, or 1 week. In the deep-encoding condition, their participants first performed cued recall, and then performed picture matching, word jumble, hand writing, and word shouting tasks to enhance their memory for the Probes. In contrast, the shallow-encoding group only performed cued recall and a paraphrasing task about a news story covering the mock crime. Seymour and Fraynt found that, for the deep-encoding condition, RT-CIT efficacy remained robust across all three delay timeframes. Importantly, for the shallow-encoding condition, RT-CIT accuracy decreased as the gap time increased, thus demonstrating that encoding strength does have an impact on RT-CIT accuracy.

In the context of the current task, we ensured adequate memory strength by asking our participants to go through the questionnaire (15 min), jigsaw puzzle (15 min), and a final rehearsal (5 min), plus the two pretests in the study phase before their participation in the RT-CIT. The number of tasks in this phase is quite similar to the deep encoding condition by Seymour and Fraynt (2009), which resulted in our study phase being at least 35 min. This similarity to a deep-encoding design may have contributed to the unexpected absence of the angular rotation effect from the present experiment. To this end, we conducted Experiment 2 with a much shorter and easier study phase to see whether the possible effect of photograph rotations in the RT-CIT would surface with shallow encoding.

## Experiment 2

The purpose of this experiment is to simplify the study phase to create a shallow-encoding scenario. This would in turn disambiguate the source of the results from Experiment 1: memory-based vs perception-based. Specifically, if memory strength was the driving factor for the absence of an angle effect in Experiment 1, the manipulation of shallow encoding in the present experiment should change the shape or slope of the RT distribution for Probes across all angles (i.e., same RT for Probe and Irrelevant at certain angles). Alternatively, if memory strength was not responsible for the results from Experiment 1, then we should observe the same results here even with the shallow-encoding study phase. To this end, in this experiment we have eliminated the 15-min questionnaire and 15-min jigsaw puzzle task from the study phase, leaving only the flashcard rehearsal and memory pretest.

### Methods

#### Participants

Twenty-nine participants (11 male, 18 female; age 20–29 years, mean age = 21.52) with normal eyesight or corrected-to-normal eyesight were recruited for this experiment. All participants gave informed consent prior to their participation, and received financial compensation for their time. Three participants’ data were excluded from analysis because the participants did not follow the instruction (i.e., Probe accuracy below 50%). All experimental procedures were approved by the Joint Institutional Review Board of Taipei Medical University, Taiwan.

#### Materials and procedure

All apparatus and materials were identical to those from Experiment 1. The only difference was the structure of the study phase. In this experiment, participants skipped the 15-min questionnaire and 15-min jigsaw puzzle and went straight to the 5-min flashcard rehearsal. After the flashcard rehearsal, participants took the computerized 4-min memory pretest and were allowed to take the pretest again if their accuracy fell under 85%. After that, participants were allowed to enter the formal RT-CIT session.

### Results

**Fig. 6:**
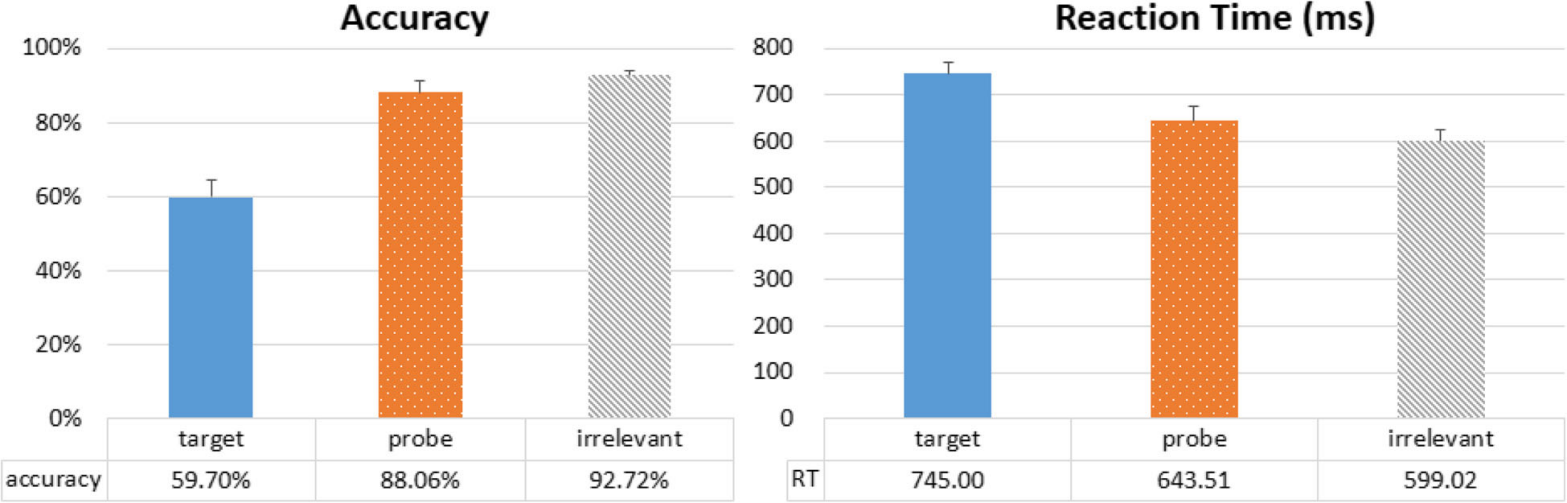
Overall accuracy and reaction time in the shallow-encoding condition. Probe item accuracy was lower than that of Irrelevant items, and the reaction time was slower

We performed the same analyses as in Experiment 1, and error trials with incorrect or no responses were discarded from analysis of RT data. First, collapsed RT data were submitted to a repeated-measures one-way ANOVA (Probe, Target, Irrelevant), and we observed a significant main effect of trial type, *F*(2,50) = 146.475, *p* < 0.001, η^2^_p_ = 0.854. Subsequent post-hoc comparisons using Bonferroni correction revealed significant differences between RT from all trial types: Target vs Probe, *t(*25) = 10.054, *p* < 0.001, *d* = 1.972; Target vs Irrelevant, *t*(25) = 16.124, *p* < 0.001, *d* = 3.162; and Probe vs Irrelevant, *t*(25) = 6.598, *p* < 0.001, *d* = 1.294 (Fig. 6, right). Accuracy data were also submitted to a repeated-measures one-way ANOVA, and revealed a significant main effect of trial type, *F*(2,50) = 106.755, *p* < 0.001, η^2^_p_ = 0.810, where post-hoc comparisons with Bonferroni correction also showed significant differences between all trial types: Target vs Probe, *t*(25) = − 9.881, *p* < 0.001, *d* = − 1.938; Target vs Irrelevant, *t*(25) = − 12.320, *p* < 0.001, *d* = − 2.416; and Probe vs Irrelevant, *t*(25) = − 2.928, *p* = 0.022, *d* = − 0.574 (Fig. 6, left). These results are very similar to those from Experiment 1.

#### Effect of study-test angular deviations in shallow encoding

**Fig. 7:**
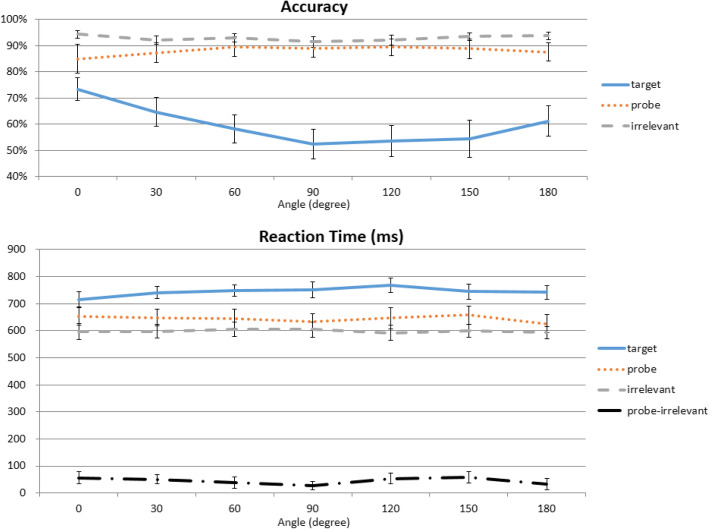
Effect of angular deviations in the shallow-encoding condition. Participants were tested on the same (0°) or rotated versions of what they saw (30°–330°, symmetrically collapsed into 30°–180° here). There is a slight dip in reaction time (RT) at 90°, but this did not reach statistical significance. No matter how big the angular deviation, separate comparisons showed that Probe RTs are slower than Irrelevant RTs at all angles. This is similar to what was observed in Experiment 1

The accuracy and RT data were then broken down by degrees of rotation. In accuracy, a repeated-measures two-way ANOVA with the factors of trial type (Probe, Target, Irrelevant) and angular deviation (0°, 30°, 60°, 90°, 120°, 150°, 180°) revealed a main effect of both trial type, *F*(2,50) = 108.617, *p* < 0.001, η^2^_p_ = 0.813, and deviation, *F*(6,150) = 8.698, *p* < 0.001, η^2^_p_ = 0.258, and a significant interaction between the two, *F*(12,300) = 11.958, *p* < 0.001, η^2^_p_ = 0.324. Subsequent one-way ANOVAs showed that there were significant effects of angular deviations for Targets, *F*(6,150) = 16.655, *p* < 0.001, η^2^_p_ = 0.400, and Irrelevants, *F*(6,150) = 3.325, *p* = 0.014, η^2^_p_ = 0.117, but not for Probes, *F*(6,150) = 1.462, *p* = 0.219, η^2^_p_ = 0.055 (Fig. 7, top panel).

RT data were submitted to a 3 × 7 repeated-measures ANOVA, which showed significant effects of trial type, *F*(2,50) = 145.908, *p* < 0.001, η^2^_p_ = 0.854, and interaction between trial type and deviation, *F*(12,300) = 3.692, *p* < 0.001, η^2^_p_ = 0.129, but no effect of angular deviation, *F*(6,150) = 1.823, *p* = 0.098, η^2^_p_ = 0.068. Using Bayesian repeated-measures ANOVA, there was a significant main effect of trial type, reflected in a higher Bayes factor for the alternative hypothesis than the null hypothesis (BF_10_ = 4.239 × 10^126^). This was not true for angular deviation, as the Bayes factor was less than 1 (BF_10_ = 0.003). For the interaction, we compared the BF_10_ value between models with and without interaction, and obtained a Bayes factor of 1.686, which falls in the “anecdotal evidence” range (Wagenmakers et al., 2018).

Subsequent one-way ANOVAs showed that there were significant effects of angular deviations for Targets, *F*(6,150) = 4.257, *p* = 0.001, η^2^_p_ = 0.146, marginally significant effects for Probes, *F*(6,150) = 2.128, *p* = 0.053, η^2^_p_ = 0.078, but not significant effects for Irrelevants, *F*(6,150) = 1.361, *p* = 0.234, η^2^_p_ = 0.052. The marginally significant effect in Probes is worth noting, as this trend was not observed at all from Probes in Experiment 1, *F*(6,138) = 0.857, *p* = 0.561, η^2^_p_ = 0.036. Although not yet statistically significant, this seems to suggest that when memory strength is low, the potential impact of rotation, if any, can possibly become slightly more influential. However, despite the marginally significant main effect of angular deviation, the one-way repeated-measure ANOVA of the Probe–Irrelevant RT difference failed to show statistical significance, *F*(6,168) = 1.507, *p* = 0.179, η^2^_p_ = 0.051 (Fig. 7). Probe RTs were still significantly slower than their Irrelevant counterparts at every angle in *t* tests with Bonferroni correction (0°, *t*(25) = 4.827, *p* < 0.001, *d* = 0.947; 30°, *t*(25) = 5.836, *p* < 0.001, *d* = 1.145; 60°, *t*(25) = 3.509, *p* = 0.012, *d* = 0.688; 90°, *t*(25) = 3.200, *p* = 0.026, *d* = 0.628; 120°, *t*(25) = 5.188, *p* < 0.001, *d* = 1.017; 150°, *t*(25) = 5.525, *p* < 0.001, *d* = 1.084; 180°, *t*(25) = 3.200, *p* = 0.026, *d* = 0.628 (Bonferroni correction)) (Fig. 7, bottom panel). Therefore, we have successfully replicated the results from Experiment 1, and the RT-CIT seems uncompromised with shallow encoding.

#### Presentation angle

A two-way repeated-measures ANOVA with the factors of trial type (Target, Probe, Irrelevant) and presentation angle (12 angles from 0° to 330°) was conducted for both accuracy and RT. There was a main effect for both trial type (accuracy, *F*(2,50) = 110.336, *p* < 0.001, η^2^_p_ = 0.815; RT, *F*(2,50) = 149.178, *p* < 0.001, η^2^_p_ = 0.856) and presentation angle (accuracy, *F*(11,275) = 12.121, *p* < 0.001, η^2^_p_ = 0.327; RT, *F*(11,275) = 4.825, *p* < 0.001, η^2^_p_ = 0.162), as well as a significant interaction between them (accuracy, *F*(22,550) = 7.475, *p* < 0.001, η^2^_p_ = 0.230; RT, *F*(22,550) = 3.943, *p* < 0.001, η^2^_p_ = 0.136).

**Fig. 8:**
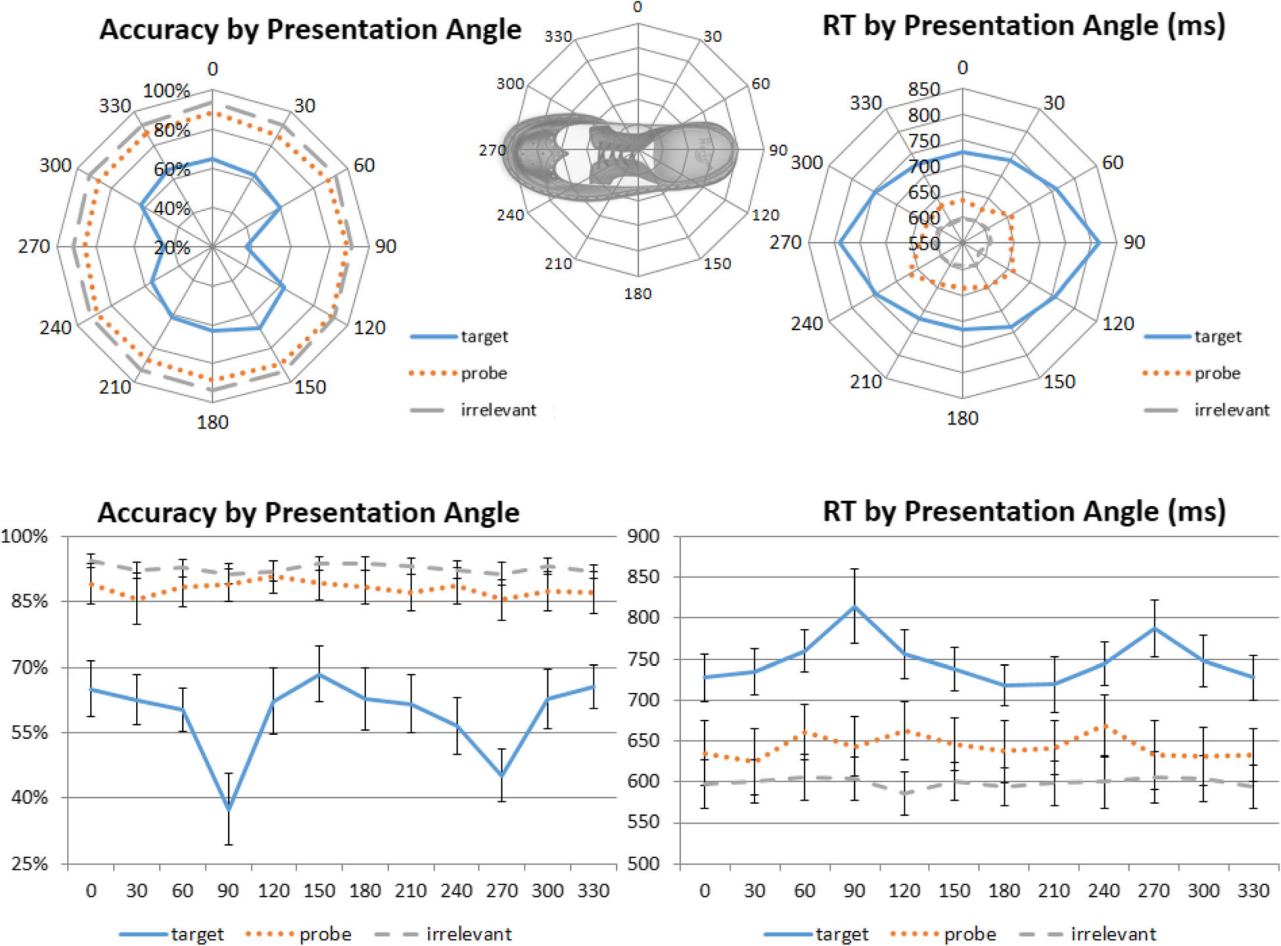
Effect of presentation angle in the shallow-encoding condition. Top panel: radar graphs depicting the effects of the presentation angles on accuracy (left) and reaction time (RT) (right). Lower panel: like Experiment 1, a strong presentation angle effect in Targets and a mild effect in Irrelevants, but no effect in Probes

Similar to Experiment 1, in accuracy, a quartic trend was significant for Targets, *F*(1,25) = 23.061, *p* < 0.001, η^2^_p_ = 0.480, but not for Probes, *F*(1,25) = 0.911, *p* = 0.349, η^2^_p_ = 0.035, and Irrelevants, *F*(1,25) = 2.739, *p* = 0.110, η^2^_p_ = 0.099. For RT, significant quartic trends were observed for both Target items, *F*(1,25) =3 0.063, *p* < 0.001, η^2^_p_ = 0.546, and Irrelevant items, *F*(1,25) = 5.045, *p* = 0.034, η^2^_p_ = 0.168, but not for Probes, *F*(1,25) = 0.137, *p* = 0.714, η^2^_p_ = 0.005 (Fig. 8). These results are pretty much identical to the results from Experiment 1, suggesting that the effect of presentation angle is robust but also not dependent on memory strength.

#### RT-CIT efficacy over time in shallow encoding

**Fig. 9:**
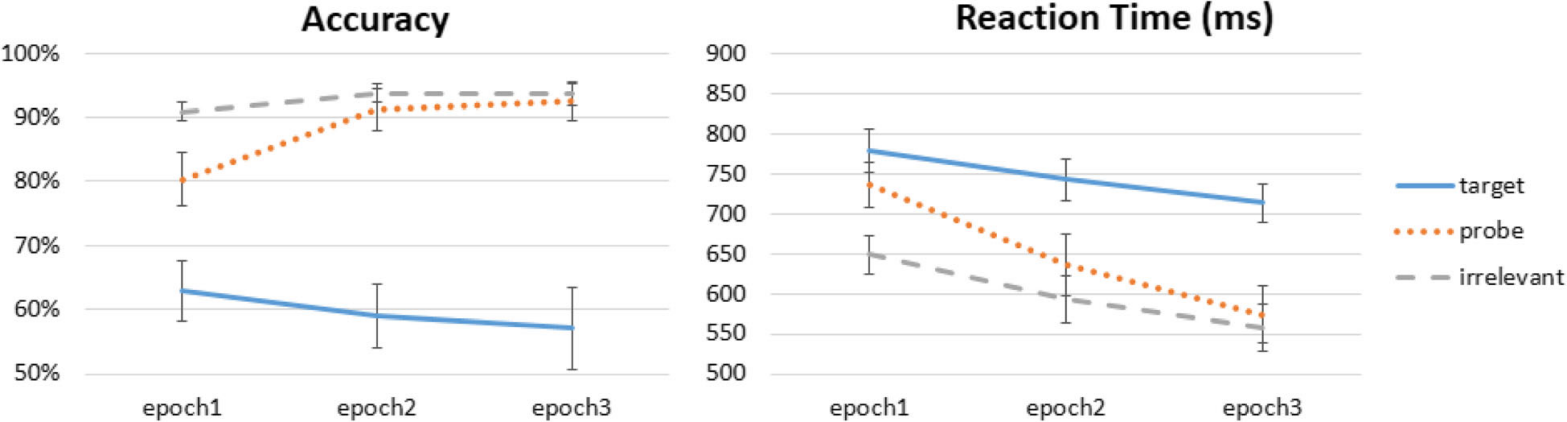
RT-CIT efficacy over time with shallow-encoding. Like Experiment 1, Probe accuracy was only significantly lower than Irrelevants in epoch 1. Unlike Experiment 1, Probe reaction time was not significantly slower than Irrelevants in epoch 3, suggesting a faster decrease in reaction time-based Concealed Information Test (RT-CIT) efficacy with shallow encoding

Trials were divided into three epochs as in Experiment 1, and a repeated-measures ANOVA on RT data with factors of trial type (Probe, Target, Irrelevant) and epoch (1, 2, 3) revealed main effects for both epoch, *F*(2,50) = 67.304, *p* < 0.001, η^2^_p_ = 0.729, and trial type, *F*(2,50) = 152.793, *p* < 0.001, η^2^_p_ = 0.859, as well as a significant interaction between them, *F*(4,100) = 22.714, *p* < 0.001, η^2^_p_ = 0.476. Post-hoc analyses with Bonferroni correction suggest that the interaction was driven by the significant gap between the Probe and Irrelevant RT from the first and second, but not third, epoch (first, *t*(25) = 9.176, *p* < 0.001, *d* = 1.800; second, *t*(25) = 4.910, *p* < 0.001, *d* = 0.963; third, *t*(25) = 2.356, *p* = 0.080, *d* = 0.462; Fig. 9, right). In comparison with Experiment 1, where all three epochs showed significant difference between Probes and Irrelevants, findings here suggest that shallow encoding indeed can compromise RT-CIT efficacy over time, which is consistent with Seymour and Fraynt’s (2009) findings. Note that this is not to suggest that fewer trials are better. On the contrary, the Probe–Irrelevant difference in epoch 3 was still marginally significant, and more data could increase statistical power and improve detection accuracy. We discuss this point further in the General discussion.

Accuracy data submitted to a two-way ANOVA showed a main effect of trial type and epoch (1, 2, 3), and a significant interaction between them: type, *F*(2,50) = 105.083, *p* < 0.001, η^2^_p_ = 0.808; epoch, *F*(2,50) = 9.204, *p* = 0.001, η^2^_p_ = 0.269; and interaction, *F*(4,100) = 15.697, *p* < 0.001, η^2^_p_ = 0.386; Fig. 9, left). Post-hoc analyses with Bonferroni correction showed that Probe accuracy went from ~ 80% in the first epoch to ~ 91% in the second epoch and ~ 92% in the third epoch (first, *t*(25) = − 4.942, *p* < 0.001, *d* = − 0.969; second, *t*(25) = − 1.531, *p* = 0.415, *d* = − 0.300; third, *t*(25) = − 0.831, *p* = 1.000, *d* = − 0.163). Therefore, unlike the robust effect in RT, there was no difference between Probe and Irrelevant accuracy after the first ~ 250 trials, which is similar to Experiment 1.

## General discussion

In this study we investigated whether angle differences between RT-CIT photographs and participants’ actual exposure and memory would have an impact on RT-CIT validity. We manipulated the deviation angles, from 0° to 330° in 11 steps, between the pictures that were seen initially and the ones that were eventually used for testing. Together, our main findings are as follows: we could not detect an effect of angular rotation in Probes; despite this, the Probes are significantly slower than the Irrelevants at all angular deviations; there is a significant Presentation angle effect at 90° and 270°; and the Probe–Irrelevant difference decreased over time, but remained statistically significant for RT difference (between Probes and Irrelevants) at almost 800 trials in Experiment 1 (deep encoding) and around 500 trials in Experiment 2 (shallow encoding).

### Angular rotations and memory strength in the RT-CIT

Our results here suggest a robust recognition memory effect in the RT-CIT against varying degrees of rotations. However, this presents a stark contrast to some of the classic findings in mental rotation (e.g., Shepard & Metzler, 1971) and object recognition (Tarr et al., 1998). We suspected that this may have been due to the deep-encoding procedure from Experiment 1, where although participants were only exposed to one angle, they did perform a total of three tasks (i.e., questionnaire, jigsaw puzzle, and computerized test) to ensure deep encoding of that particular angle. To this end, we conducted Experiment 2 using a shallow-encoding study phase. However, results from Experiment 2 very much mimicked those from Experiment 1. There was a slight dip in the Probe RT (Fig. 7, bottom panel) under shallow encoding, but even so the Probe–Irrelevant difference was still significant.

The lack of a detectable effect of encoding strength on rotational angles may seem surprising at first. However, it is important to note that our study here can be equated with the 10-min delay condition in the Seymour and Fraynt (2009) study. In their study, deep and shallow encoders were both easily classified by the RT-CIT in the 10-min delay condition, suggesting the RT-CIT’s effectiveness in detecting fresh memory traces even for shallow-encoded words. RT-CIT efficacy was only compromised after a 24-h or 1-week delay for shallow encoders. Our results are consistent with the 10-min delay condition, and from their study we would infer that our Experiment 2 would show lower detectability after a longer time delay due to shallow encoding of the pictures. This is somewhat similar to the literature on central vs peripheral items and their effects on CIT efficacy (Carmel, Dayan, Naveh, Raveh, & Ben-Shakhar, 2003; Gamer, Kosiol, & Vossel, 2010). Carmel et al. (2003) found that using peripheral items from the mock crime would decrease CIT efficacy, and Gamer et al. (2010) further clarified that this is true for both culprits and informed innocents (using a self-referential version of the CIT called the Guilty Action Test), but the use of central Probe items is able to separate culprits from informed innocents after a 2-week delay, presumably because culprits had deeper encoding of the central items than the innocents. In addition to encoding strength, research has shown that encoding type can also affect the outcome of the RT-CIT. Recently, Geven, Ben-Shakhar, Kindt, and Verschuere (2019) presented their participants with crime-related information at either the categorical level (e.g., car) or the exemplar level (e.g., Volkswagen) during the encoding stage, and tested the participants with either congruent (e.g., categorical–categorical, exemplar–exemplar) or incongruent (e.g., categorical–exemplar, exemplar–categorical) information in the RT-CIT. These authors found that RT-CIT efficacy decreased when mismatched Probes were used, and adding a 1-week delay between the study and test phases did not change the outcome. Although our null results here seem to be contradictory to Geven et al.’s findings, such discrepancy can probably be attributed to the mode of presentation (i.e., text vs images). Nevertheless, future research on the possible interaction between angular rotations, encoding strength, encoding type, and time delay (i.e., memory decay) is definitely warranted.

### Object representations: viewpoint-dependent vs viewpoint-invariant

The results from Experiment 2 suggest that the lack of an angular rotation effect was not due to encoding strength. This implies that the results may have been driven by earlier, perceptual, processes. As previously mentioned, most classic findings in object recognition have implicated an element of viewpoint-dependence, where the RT would increase with angle rotation in 3D objects (Jolicoeur et al., 1985), geons (Tarr et al., 1998), specific subcategorical animals or objects (Hamm & McMullen, 1998; Milivojevic, 2012), and even familiar items (Jolicoeur, 1985, 1990). Therefore, it is quite surprising that our Probe and Irrelevant RT differences in the RT-CIT would remain unaffected regardless of the degree of angle deviations. There are two possible reasons for this.

First, mental rotation studies usually use an unpredictable rotational axis, whereas the axis is fixed in the present study. This point is of particular interest since our data also suggest a strong angular effect in *presentation* angles (Fig. 4). In fact, if anything, the effect of presentation angle on Probe RT is stronger than study-test deviation angles, suggesting that the presentation of the Probe images is perhaps a more important factor that law enforcement should consider. This is consistent with most perception research that demonstrates how people prefer off-axis (3/4) canonical views of objects, most likely due to the fact that these views display more surfaces, and thus facilitate object identification (Gardony, Taylor, & Brunyé, 2014; Palmer, Rosch, & Chase, 1981; Gómez, Shutter, & Rouder, 2008). In the context of the CIT, although object rotation was never investigated, Ben-Shakhar and Gati (1987, 1990, 1992, 2003; Ben-Shakhar, Gati, & Salamon, 1995; Ben-Shakhar, Gati, Ben-Bassat, & Sniper, 2000; Gati, Ben-Shakhar, & Avni-Liberty, 1996) did conduct a series of studies that examined the degree of similarity between study-test items (verbal and pictorial) and its effect on participants’ skin conductance responses. In general, these authors found that participants’ electrodermal responses varied systematically with the differing degrees of study-test similarity, which suggests that electrodermal responses to CIT are not an all-or-none process; rather, they varied in a systematic way that was resemblant to a feature-by-feature matching process (Gati & Ben-Shakhar, 1990). In the present study, although we used RT measures and did not completely add or delete any significant or novel features, our rotation manipulation did change the amount of visible areas of certain features (e.g., stripes on the side), which can also be understood as varying degrees of feature occlusion. In this light, our findings in the presentation angle analysis, where RTs varied monotonically with varying degrees of feature rotation, can perhaps be interpreted as varying degrees of feature occlusion, with 90° and 270° being the maximal occlusion condition. Note that the stronger effect of presentation angle at 90° and 270° here is closely associated with the stimuli being used, thus it is important to determine the acceptable range of presentation angle on a stimulus-specific basis. But all in all, although the study-test deviation angle does not matter, our data suggest that obscure *presentation* angles that make object identification harder should be avoided.

Second, laboratory studies often use highly similar and colorless items that differ by only one subtle feature, whereas the images used here are real-life objects with more featural clues for object identification. Therefore, although we have used multiple stimuli that share the same shape, color, and functional category, these real-life photographs are nonetheless still more colorful and geometrically dissimilar than line drawings and sketches that are often used in the laboratory. Indeed, one study by Hochberg and Gellman (1977) in the mental rotation literature has found that salient and unique “landmark” features can almost flatten mental rotating time. This is consistent with our findings here, which is likely the key factor that drove the flat slope in the study-test deviation condition. In addition, the stimuli we have chosen here are everyday objects that people have become experts of. That is, much like face recognition, shoe discrimination is also a process that operates at the subordinate level (e.g., this is a Nike running shoe, this is Jill’s face, etc.) as opposed to the exemplar level (e.g., this is a shoe, this is a face, etc.). Therefore, in real-life photographs, subtle differences can be salient enough to facilitate rapid and automatic recognition. To this end, it would be interesting to see whether the effect of angular rotations would begin to surface and affect RT-CIT efficacy in non-expert Probe items where people cannot easily differentiate subordinate identities (e.g., different models or makes of a pistol).

One observation of theoretical importance to note is that, in the present experiment, many stimuli were matched via different featural dimensions (e.g., shape or color). Consequently, paying attention to any one single feature will not be able to produce the level of object recognition observed in the present study—because there are always multiple other shoes of similar shape or color. This suggests an automatic activation of holistic, or “bound”, object representation from long-term memory that is the result of rapid feature-binding across multiple featural dimensions (e.g., shape, color) instead of just any one feature alone. Indeed, one recent study by Tarr and Hayward (2017) suggests that perhaps object constancy is supported by recognition of both global and local features that are processed via different brain regions (Gauthier et al., 2002; Lescroart & Biederman, 2013), hence why object recognition can be both viewpoint-dependent (i.e., local features) and viewpoint-invariant (i.e., global features) by combing multiple sources of visual information. The present results support this hybrid model of viewpoint-dependence and viewpoint-invariance, and further suggest that the rapid processing of global and local features plus the automatic activation of bound representations can be advantageous in increasing the robustness of the RT-CIT against variances in viewpoints.

### RT-CIT sensitivity over time

To assess RT-CIT validity over trial progression, we separated the 760 trials into 3 epochs to analyze the magnitude of the delay between Probe and Irrelevant trials. Although the slowing of Probe RT remained statistically significant in all three epochs, it is quite clear that the size of the effect decreased over time (Fig. 5, right panel). Since each intervening deviation angle between 0° and 180° in either direction was only used once throughout the entire experiment, this minimization of the slowing RT cannot be explained by repeated exposure of the same Probe image. Rather, this decrease in the gap between Probes and Irrelevants should be attributed to the slowing RT of the Irrelevant stimuli, which suggests that the participants were starting to build a more robust representation of the Irrelevant stimuli via mere exposure of the same item at other angles. In real-life scenarios, if we consider additional factors often associated with time, such as memory contamination (Wixted, Mickes, Clark, Gronlund, & Roediger III, 2015; Wixted & Wells, 2017) or verbal descriptions that are known to “overshadow” visual memory (Mickes & Wixted, 2015; Wilson, Seale-Carlisle, & Mickes, 2017), the statistically significant gap in the third epoch is likely an optimistic estimate. But realistically speaking, even the trial number from two epochs should be more than enough for field investigations, assuming that the Probe items have strong memory strength. In this light, our results here suggest that the Irrelevant stimuli can be used in the form of different angles for at least 500 trials and remain effective, with a more robust effect from the first bin (i.e., first 250 trials).

The decrease in Probe–Irrelevant difference over time reported here may seem contradictory to previous findings in the literature. For example, Kleinberg and Verschuere (2015) used a web-based RT-CIT on a large sample and found that the effect of the RT-CIT was actually more valid and reliable over time, so much so that the authors recommended at least 200 trials for optimized RT-CIT performance (also see Verschuere & Kleinberg, 2016). It is important to note, however, that although the Probe–Irrelevant difference decreased over time, it remained statistically significant in all three epochs for deep-encoders, and was marginally significant in the third epoch for shallow-encoders. Furthermore, it is possible that the additional epoch can still improve the overall RT-CIT accuracy by adding more data. Thus, although the effect becomes smaller with more trials, the predictive power of the RT-CIT would nonetheless increase.^4^ On another relevant note, the total length from the (Kleinberg and Verschuere 2015) study was 360 trials, which is about half of our experiment length (i.e., 760 trials). Therefore, the longer test length that these authors have recommended (i.e., 200 trials) is also well within our recommended range (i.e., 250 trials). In fact, if anything, the two studies are quite consistent with each other in the range for the number of recommended trials, and the more prominent difference is actually the measurement (Probe–Irrelevant difference vs area under the curve), which can be informative for future studies.

## Conclusions and practical implications

Given the potential legal impact of the RT-CIT, and to prevent misuse of this tool, all protocols and designs of the RT-CIT should be evidence-based and thoroughly investigated before permissibility in court. To this end, the current findings have several implications for future designs of RT-CIT materials: if the encoding strength is strong (e.g., repeated or long exposure, exposure via multiple modality, etc.), the forensic team does not have to worry too much about reconstructing the angles that match what the perpetrators saw—but unnatural angles such as 90° and 270° or an unconventional rotational axis were more difficult to detect than others, and thus field officers may wish to consider avoiding those angles in forensic contexts; and in the context where angles are randomized and do not match the suspects’ memory, as in the current study, it is perhaps safer to achieve at least 250 trials to stabilize and improve RT-CIT efficacy.

## Supplementary information

**Additional file 1: Supplementary Figure.** Twenty Target and Probe images from this experiment.

## Data Availability

The dataset from the current study is currently not publicly available because our participants did not choose to opt in on data sharing on the consent form. However, we are working on the re-consenting procedure and expect to obtain approval to share the data soon.

## Abbreviations

RT: Reaction time
CIT: Concealed Information Test
RT-CIT: Reaction time-based Concealed Information Test
EEG: Electroencephalogram

## Footnotes

1 The stimuli and e-prime code of this study are available for download at https://osf.io/45j2n/.

2 One repeating trial was added for 0° and 180° because these two angles only have half the trial number of other angular deviations. That is, if a participant studied a 0° picture in the study phase, then a deviation of 30° can come from presentation angles of either 30° or 330°, because the item can rotate toward the left (30°) or the right (330°). This was not the case for 0° and 180°, so one repeating trial was added for these angles to equate the trial numbers under each condition.

3 De-identified data from participants who consented to the sharing of their data are securely stored on a private repository (https://osf.io/vc8d2/) and are available on request for further analysis.

4 We thank one anonymous reviewer for bringing up this point.
