# Supplementary material for: Variation of picture angles and its effect on the Concealed Information Test
Source: Cogn Res Princ Implic. 2020 Jul 31;5:33. doi: 10.1186/s41235-020-00233-6 (PMC7394988; doi:10.1186/s41235-020-00233-6)
Supplement: Supplementary file 1 — Additional file 1: Supplementary Figure. Twenty Target and Probe images from this experiment. [file 41235_2020_233_MOESM1_ESM.docx]

Supplementary Figure


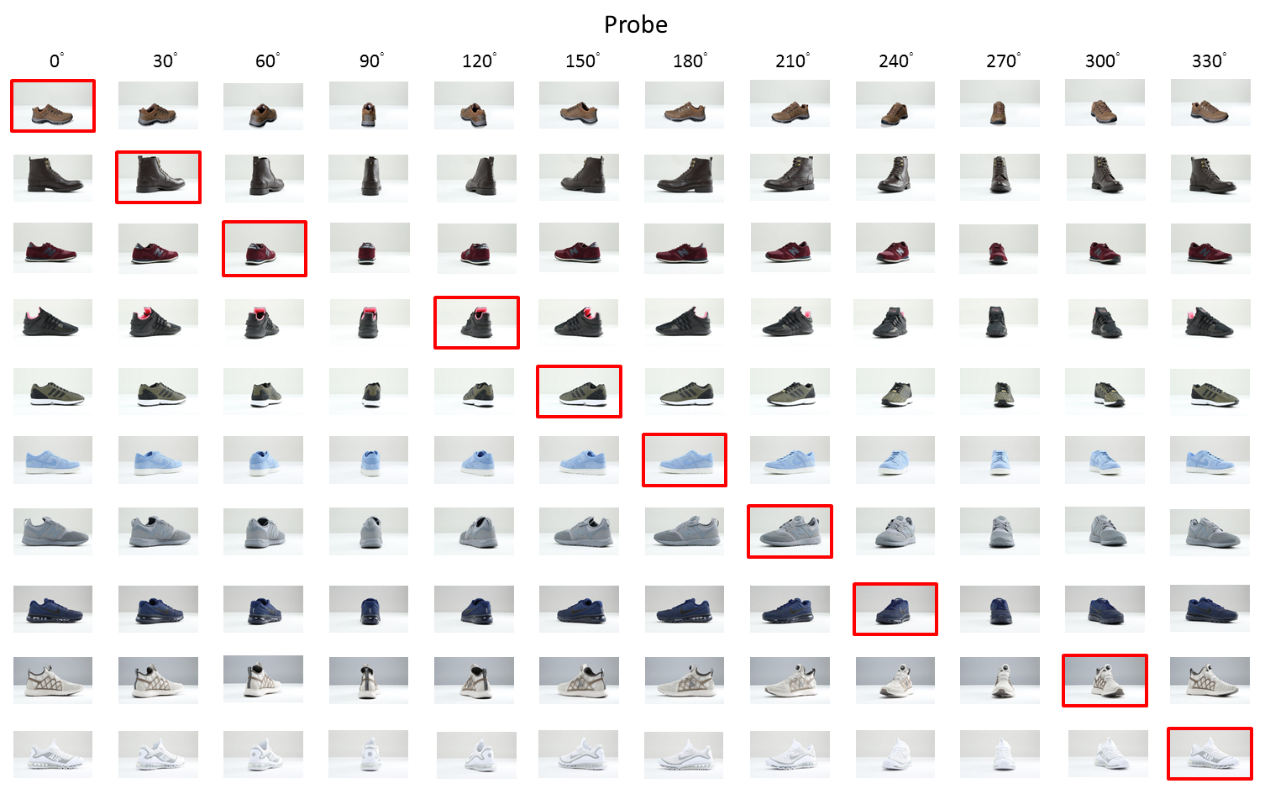


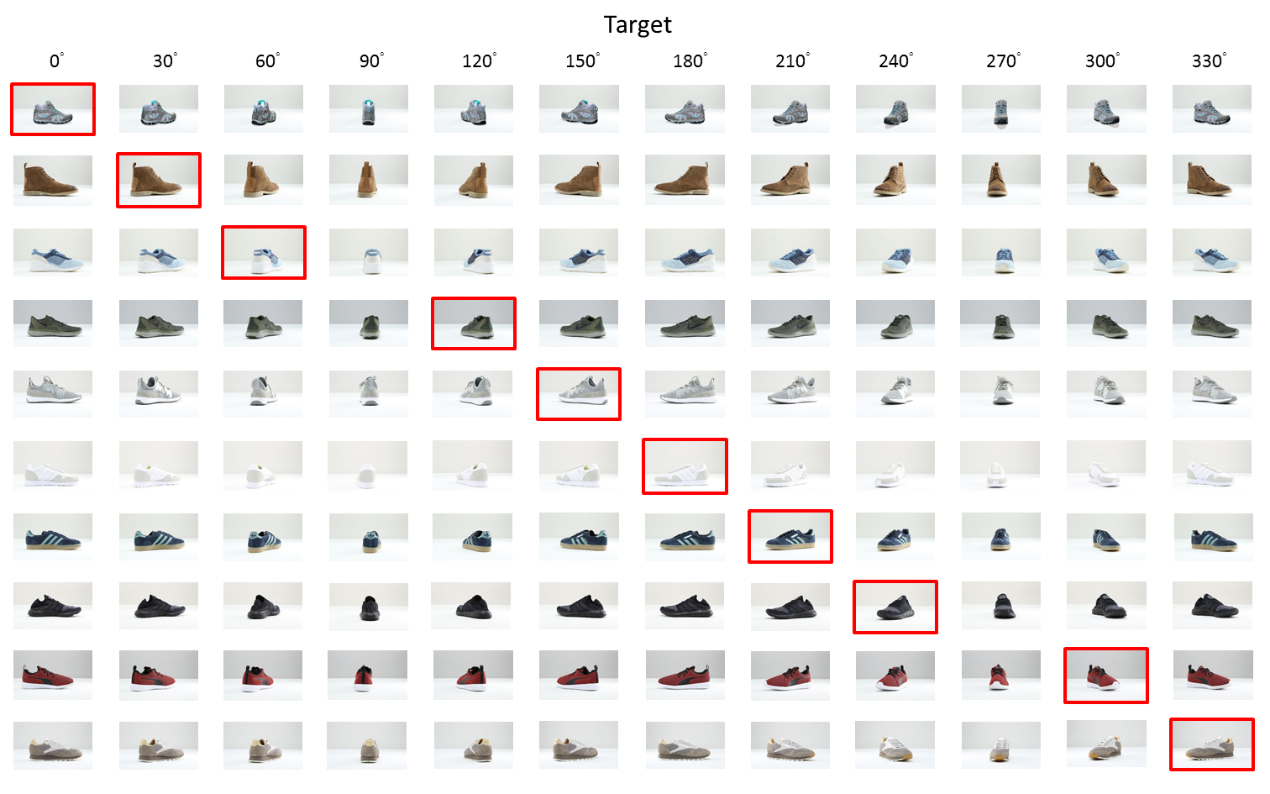
 Twenty Target and Probe images from this experiment. Target and Probe images were matched in terms of color, shape, and function/category (e.g., a blue sneaker in Probe will also have a similarly-shaped blue sneaker in Target). Ninety and 270 degree images were not used for the study phase, and were only used during the test phase. Images enclosed in red were ones used in study phase, and were also used in the test phase with all other unseen angles, plus the 480 Irrelevants.
